# A mouse model of vitamin D insufficiency: is there a relationship between 25(OH) vitamin D levels and obesity?

**DOI:** 10.1186/s12986-017-0174-6

**Published:** 2017-03-11

**Authors:** Kenneth L. Seldeen, Manhui Pang, Maria Rodríguez-Gonzalez, Mireya Hernandez, Zachary Sheridan, Ping Yu, Bruce R. Troen

**Affiliations:** Division of Geriatrics and Palliative Medicine, Department of Medicine, Jacobs School of Medicine and Biomedical Sciences, University at Buffalo and Research Service, Veterans Affairs Western New York Healthcare System, Buffalo, NY USA

**Keywords:** Vitamin D, PTH, Insufficiency, Obesity, Weight, BMI, Body Composition, Mice, Fat, High fat diet

## Abstract

**Background:**

Vitamin D insufficiency (serum 25-OH vitamin D > 10 ng/ml and < 30 ng/ml) is prevalent in the obese (body mass index (BMI) > 30 kg/m^2^), yet relationships between the two are poorly understood. Objectives of this study include identification of the impact of obesity on reducing serum 25-OH vitamin D concentration, particularly in response to altered vitamin D_3_ supplementation, and to elucidate the longitudinal impact of serum 25-OH vitamin D on body mass index.

**Methods:**

Twenty four-week-old lean and obese male C57BL/6 J mice were fed low, standard, or high levels of cholecalciferol supplementation and followed for 24 weeks. Longitudinal measurements include serum 25-OH and 1,25-(OH)_2_ vitamin D, intact PTH, and calcium concentrations, as well as BMI, bone density and body fat/lean mass.

**Results:**

Baseline serum 25-OH concentrations were not different in lean and obese mice (lean 32.8 ± 4.4 ng/ml versus obese 30.9 ± 1.6 ng/ml p = 0.09). Lean mice receiving low supplementation exhibited rapid declines in serum 25-OH vitamin D concentrations, falling from 33.4 ± 5.4 ng/ml to 14.5 ± 3.4 ng/ml after 2 weeks, while obese mice declined at a lower rate, falling from 30.9 ± 1.5 to 19.0 ± 0.9 ng/ml within the same time period. Surprisingly, high vitamin D_3_ supplementation did not substantially increase serum vitamin D concentrations above standard supplementation, in either lean or obese mice. No differences in serum 1,25-(OH)_2_ vitamin D, intact parathyroid hormone (PTH) or serum calcium were observed between lean and obese mice within the same vitamin D supplementation group. Yet obese mice exhibited lower serum calcitriol, higher serum PTH, and lower bone mineral density (BMD) than did lean mice. Additionally, neither body mass index nor body fat % was significantly correlated with vitamin D concentrations. Interestingly, lean mice with high vitamin D supplementation consumed significantly more food than did lean mice with standard or low supplementation (14.6 ± 1.7 kcal/mouse/day versus 11.8 ± 1.4 and 12.3 ± 1.7 respectively, *p* < 0.0001 for both).

**Conclusions:**

Low cholecalciferol supplementation in both lean and obese mice significantly and sustainably reduces serum 25-OH vitamin D concentrations. Interestingly, obesity slowed the rate of decline. Over the period of the study, vitamin D insufficiency was not subsequently correlated with greater BMI/body fat, although lean mice with high supplementation consumed greater calories with no apparent BMI increase.

## Background

Vitamin D insufficiency (serum 25-OH vitamin D >10 ng/ml and <30 ng/ml) is a prevalent condition with an estimated 70% of the population considered at risk [1, 2]. Concurrent with widespread vitamin D insufficiency, the Centers for Disease Control also reports that a large percentage of the population (>35%) has a body mass index (BMI) considered obese. Several human studies have explored the possibility that these two phenomena may be linked [3–6], however, it remains unclear if vitamin D insufficiency promotes weight gain and/or if obesity modulates serum vitamin D concentration. Interestingly, studies seeking to modulate body weight by correcting vitamin D status have been unsuccessful [7–9], although other beneficial effects, including reduced body fat [7], reduced inflammatory profile [9], and improved insulin resistance [10] were seen. Contrary to what is seen in human trials, animal studies appear to show that vitamin D may promote weight gain. Vitamin D receptor (VDR) knock-out mice were shown to be resistant to weight gain [11, 12], as were diet-induced vitamin D deficient/insufficient mice to high fat [13] and “western” diets [14]. Additionally, over-expression of VDR in adipocytes induced weight gain in mice [15]. Although interpretation of these data suggests that vitamin D, through VDR, increases weight gain, other studies have shown that administration of 1,25-(OH)_2_ vitamin D, the active form of vitamin D and direct ligand to VDR, decreases weight and has other beneficial effects [16, 17].

Alternatively, data from several studies suggest obesity may impair the effectiveness of vitamin D supplementation. Supplementation in the obese has resulted in less than expected supplementation, [18–21] and these findings are further supported by a meta-analysis of greater than 12,000 patients showing that BMI negatively correlates with the effect of supplementation [22]. Additionally, mice given 2,000 IU vitamin D_3_/kg chow in a “western” diet had similar serum 25-OH vitamin D concentration as their lean counterparts given 1,000 IU vitamin D_3_/kg chow at the conclusion of the study [14]. It has been speculated that fat sequestration of vitamin D leads to lower serum concentrations [21], although this traditional view has recently been challenged by one study suggesting dilution by greater body mass and not fat sequestration leads to the dampening effects [23].

Studies examining the interrelationships between serum vitamin D insufficiency and obesity frequently focus on human populations where differing lifestyle and/or genetic factors may obfuscate findings. Animal models, on the other hand, predominately make use of receptor knockouts or complete dietary removal to simulate vitamin D deficient conditions, which may not accurately reflect the more prevalent condition of insufficiency in humans. To further explore the relationships between 25-OH vitamin D status and weight/body fat, we induced vitamin D insufficiency in lean and obese mice and examined food consumption, body weight, and body composition. Our data indicate that higher BMI modestly buffers serum 25-OH vitamin D concentration in response to lower supplementation. Further, our findings also suggest that neither high or low vitamin D_3_ supplementation modulates body weight or body fat.

## Methods

### Mice

All studies and experimental protocols were approved by and in compliance with guidelines of the Miami VA Animal Care and Use Committee. At all times mice were provided *ad libitum* access to chow/water and covered to avoid exposure to facility lighting. 8-week-old male C57BL/6 mice were randomly assigned to either high fat (60% FDC, Dyets Inc, Bethlehem, PA, ID# D180988) or low fat (10% FDC, ID# D180989) feeding for 16 weeks. Mice failing to gain more than 10% body weight were removed from the study. At 24 weeks of age, lean mice were randomly assigned to low fat diets containing 125, 1000 or 4000 IU vitamin D_3_/kg chow and obese mice to high fat diets containing 162, 1282 or 5169 IU vitamin D_3_/kg chow (Table 1), and were followed for 24 additional weeks. Numbers of animals were nine for lean-low & lean-standard, eight for lean-high, six for obese-low & obese-standard, and seven for obese-high. Mice and chow from cages were weighed on a weekly basis. Additionally, mouse length was measured at the end of the study by anesthetizing mice and measuring from nose to the base of the tail. BMI was calculated as g/cm^2^ [24].

**Table 1:** Diet compositions

Component (*g/kg)*	Low fat Diet	High fat diet
Vitamin Free Casein	200	258
L-Cystine	3	4
Dyetrose	35	162
Sucrose	349	88
Cornstarch	315	0
Soybean oil	25	32
Lard	20	317
t-Butylhydroquinone (TBHQ)	0.005	0.006
Cellulose	50	65
Dicalcium Phosphate	13	17
Calcium Carbonate	5.5	7.1
Potassium Citrate	16.5	21.3
Choline Bitartrate	2	2.6
Vitamin/Mineral Mix *(Vit D free)*	20	26
Vitamin D_3_	125/1000/4000 IU/kg	162/1282/5169 IU/kg
	3.1/25.0/100.0 μg/kg	4.0/32.3/129.0 μg/kg

### Serum analysis

Blood was extracted through the sub-mandibular vein using a mouse lancet (MEDIpoint, inc., Mineola, NY.) and collected into microcentrifuge tubes. Samples were held at room temperature for 10 min to allow coagulation and then centrifuged at 13,000 RPM for 10 min to allow separation of serum. Analysis of serum was performed using specific ELISA kits for 25-OH vitamin D (ImmunoDiagnostic Systems, Inc., Scottsdale, AZ), 1,25-(OH)_2_ vitamin D (MyBioSource, San Diego, CA.) and intact PTH (MyBioSource), while colorimetric assays were used to assess calcium (Biovision, San Francisco, CA.) according to manufacturer protocols.

### Dual-energy X-Ray absorptiometry

Analysis of bone mineral density, body fat % and lean mass was performed using a Lunar PIXImus II (GE Healthcare, United Kingdom). Animals were anesthetized and then analyzed with a single scan at baseline and every 8 weeks thereafter.

### Statistics

Two-way ANOVA, followed by post hoc Tukey’s Multiple Comparisons test when applicable, was used for comparisons between all vitamin D and diet groups at each time point as well as between vitamin D groups and time points, separately for obese and lean (Ian E. Holliday, 2012, Two-Way ANOVA (v1.0.3) in Free Statistics Software (v1.1.23-r7), Office for Research Development and Education, http://www.wessa.net/rwasp_Two%20Factor%20ANOVA.wasp). An * indicates statistically significant results corresponding to * for *p* < 0.05, ** for *p* < 0.01, *** for *p* < 0.001 and **** for *p* < 0.0001. All data were screened for potential outliers using GraphPad online software (La Jolla, CA.), using a significance cut-off of 0.05. All data are presented as mean ± standard deviation.

## Results and Discussion

### Serum 25-OH vitamin D concentrations fall rapidly with low supplementation to a constant insufficient level, while greater supplementation leads to little increase; obesity tempers decline in response to low vitamin D chow

**Fig. 1:**
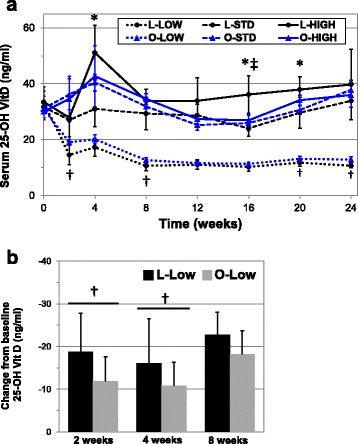
Analysis of serum 25-OH vitamin D_3_ concentrations of lean and obese mice in response to altered vitamin D_3_ supplementation. Serum 25-OH vitamin D_3_ concentration was measured every two weeks using ELISA in lean (L) and obese (O) mice given low (125 IU/kg chow), standard (STD, 1000 IU/kg chow) or high (4000 IU/kg chow) amounts of vitamin D_3_ **a** Decline from baseline serum 25-OH vitamin D concentration was further calculated for L-LOW and O-LOW **b**. Symbols indicate *p* < 0.05 between L-HIGH and L-STD (*), L-HIGH and O-HIGH (‡), and L-LOW and O-LOW (†)

In order to understand the varied interrelationships between vitamin D and body weight/composition, we set out to establish a range of serum 25-OH vitamin D concentrations between insufficiency (>10 ng/ml) and above sufficiency (>30 ng/ml) in lean and obese mice. To do so, we provided lean mice vitamin D_3_ supplementation at low (125 IU/kg chow), standard for most animal facilities (1,000 IU/kg chow) and high (4,000 IU/kg chow) amounts and in obese mice by providing low (162 IU/kg chow), standard (1,282 IU/kg chow) and high (5,169 IU/kg chow) amounts (Table 1). We increased vitamin D_3_ supplementation for obese groups to account for the lower food consumption rate that occurs during high fat feeding [25, 26]. As expected, we found no statistically significant difference in the amount of vitamin D_3_ being consumed between lean and obese mouse groups (Table 2). To confirm establishment of vitamin D insufficient and sufficient mice we measured serum 25-OH vitamin D concentrations at baseline and every two weeks thereafter (Fig. 1a). There was no statistically significant difference in serum 25-OH vitamin D between lean and obese mice (lean *n* = 26, 32.8 ± 4.4 ng/ml versus obese *n* = 19, 30.9 ± 1.6 ng/ml, *p* = 0.09). Similarly, Park et al. did not find differences in serum 25-OH vitamin D in obese and lean 22 week old mice, suggesting body composition does not modulate serum vitamin D levels during consistent supplementation [25].

**Table 2:** Mean food and vitamin D_3_ consumption in mice. Lean and obese mice were given *ad libitum* access to chow containing varying amounts of vitamin D_3_ and food was weighed weekly to determine mean (± standard deviation) consumption

Supplementation	Group	Food Consumption (g/mouse/day)	Vitamin D_3_ Consumption (IU/mouse/day)	Comparison Lean and Obese
Low	Lean	3.38 ± 0.47	0.42 ± 0.06	*p* = 1.00
	Obese	2.82 ± 0.19	0.46 ± 0.03	
Standard	Lean	3.24 ± 0.39	3.24 ± 0.39	*p* = 0.86
	Obese	2.77 ± 0.19	3.55 ± 0.25	
High	Lean	4.00 ± 0.46	15.97 ± 1.87	*p* = 0.057
	Obese	2.94 ± 0.16	15.20 ± 0.85

We then modified cholecalciferol given in chow to elucidate whether body composition affects response to altered supplementation. Mice given low supplementation experienced a rapid decline evident at two weeks, with stabilization between 11–12 ng/ml by 8 weeks, independent of being lean or obese. The rate of decline in serum 25-OH vitamin D for lean mice is consistent with findings in rats, goats and cows [27–29], but more rapid than the reported half-life of approximately 20 days in humans [30]. However, the rate of decline was steeper in lean compared to obese mice, with the decline from baseline over two weeks being 18.8 ± 5.7 ng/ml versus 11.9 ± 1.1 ng/ml (**p = 0.0084), respectively (Fig. 1b). This salient observation supports the notion that fat sequestration may buffer against the loss of serum 25-OH vitamin D during low supplement intake. Furthermore, serum 25-OH vitamin D was significantly greater at several time points in low supplemented obese mice compared to lean (week 8: *p = 0.0247, week 20: *p = 0.0410, and week 24: ***p = 0.0003, Fig. 1a), which would not be consistent with the Drincic et al. dilution model [23]. Yet, the overall magnitude of the differences (~1–2 ng/ml) does not strongly support a role of fat sequestration in controlling serum 25-OH vitamin D.

Interestingly, higher supplementation failed to significantly increase serum 25-OH vitamin D concentrations compared to standard amounts after 24 weeks in both lean and obese mice. Non-linear increases during supplementation to raise serum vitamin D above 30 ng/ml have been observed in rats [31], and similar 25-OH concentrations were detected in lean mice given 1000 IU/kg chow compared to obese mice given just over 2000 IU/kg chow [14]. Taken together, these reports and our data reflecting a non-linear increase in supplementation between 1000–4000 IU/kg chow are inconsistent with those of Fleet et al. [32] demonstrating linear increases from 400 IU/kg chow through 20,000 IU/kg chow. It is important to note that Fleet et al. used weanling mice in and did not specifically interrogate supplementation with 2000 or 4000 IU/kg chow. Furthermore, our data suggest that the obese/lean status of the mouse does not modulate the effects of higher supplementation. These findings run contrary to numerous human clinical trials showing diminished elevations with supplementation due to obesity [20, 21, 33, 34], but not all [19, 35]. A possible explanation for the lack of effect in our study was the failure of higher supplementation to increase 25-OH vitamin D levels above standard supplementation in either lean or obese mice, a ceiling effect that may be masking potential obesity impacts. Our data show that high supplemented lean mice had significantly greater 25-OH vitamin D concentration at week 16 over obese mice (week 16, **p = 0.0040 versus high supplemented obese mice), however, as this difference did not persist we were unable to confidently conclude that obesity modulates the effects of higher supplementation.

### Obese mice, independent of serum vitamin D status, exhibit differences in serum 1,25-(OH)_2_ vitamin D and intact PTH concentration relative to lean mice, with no effect on serum calcium

**Fig. 2:**
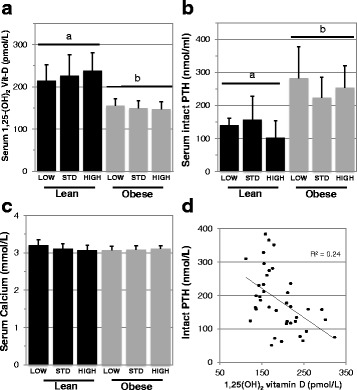
Serum profiles of lean and obese mice following supplementation with varied amounts of *Vitamin D*_*3*_. Serum was collected following 24 weeks of either low, standard or high vitamin D_3_ supplementation, and analyzed for serum 1,25-(OH)_2_ vitamin D_3_ **a**, intact PTH **b** and calcium **c** concentration using ELISA or colorimetric assay. Correlative analysis was further performed between serum intact PTH and serum 1,25-(OH)_2_ vitamin D **d**. Different letters denote a significant difference between lean and obese (****p < 0.0001)

To further identify the impacts of varied 25-OH vitamin D levels, we analyzed the serum concentration of 1,25-(OH)_2_ vitamin D, intact parathyroid hormone (PTH) and calcium (Fig. 2a-c) in lean and obese mice after 24 weeks of altered vitamin D_3_ supplementation. Our findings reveal there were no significant differences in any serum 25-OH vitamin D levels between vitamin D groups in either lean or obese mice. However, obese mice exhibited lower 1,25-(OH)_2_ vitamin D and higher intact PTH compared to lean mice (****p < 0.0001 for both), and these findings in our mouse model are consistent with data from a study in lean and obese humans [36]. We did not observe statistically significant differences in serum calcium due to obesity (*p* = 0.27). Our findings for intact PTH and serum calcium in obese and lean 48-week-old mice are in agreement with the reported levels in 22-week-old obese and lean mice [25]. However, we do report a significantly lower, and Park *et al*. [25] a significantly higher, serum 1,25-(OH)_2_ vitamin D in obese mice relative to lean. As expected, we detected a positive association between serum levels of 1,25-(OH)_2_ vitamin D and intact PTH (r^2^ = 0.24, **p = 0.0014, Fig. 2d). Whether this relationship exists in Park et al. is not reported. The nature of these conflicting data may be due to the greater length of time of exposure to high fat diet (48 weeks in our study versus 18 weeks for Park et al.) that has allowed greater time for an equilibrium between PTH and serum 1,25-(OH)_2_ vitamin D consistent with observations in human studies [36].

To our surprise, differing supplementation and the subsequent impacts on serum 25-OH vitamin D concentrations, had no significant correlation with 1,25-(OH)_2_ vitamin D or iPTH serum markers in these mice, a finding that is comparable to observations from a study in rats [31]. Although, this study may have been underpowered to detect effects on 1,25-(OH)_2_ vitamin D, intact PTH and calcium, this phenomenon may also reflect the narrow dosing range designed to induce insufficiency. Indeed effects on these serum markers were reported in other studies when supplementing outside the normo-physiological range used in our study of roughly 125–5,000 IU/kg chow [32, 37].

### Lean mice receiving higher amounts of cholecalciferol have higher caloric intake yet maintain similar body weight

**Fig. 3:**
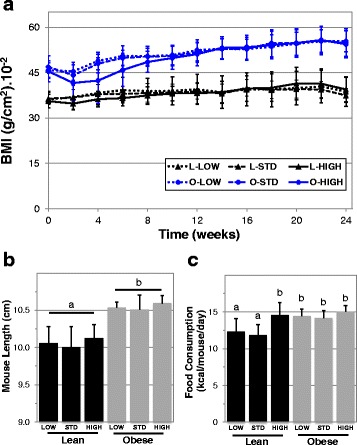
Food consumption and BMI in mice fed high or low fat diet containing varying levels of vitamin *D*_*3*_. Twenty-four week old male mice were randomly sorted into low, standard (STD) or high vitamin D_3_ supplementation groups and followed for 24 weeks. Body mass index **a** for each mouse was determined weekly using mouse length measurements from week 24 **b**. Mouse length endpoint measurements reflect the distance from nose to the base of the tail. Food consumption was measured weekly and group means were averaged **c**. Different letters denote significant differences (****p < 0.0001)

Numerous human studies have identified negative associations between body mass index (BMI) and serum 25-OH vitamin D concentrations [2, 4, 6, 38], leading to speculation that low 25-OH vitamin D may promote weight gain and correction of vitamin D status may promote weight loss in overweight individuals. To investigate the modulating effects of serum vitamin D on body mass index, we followed body weight changes in lean and obese mice supplemented with varying amounts of vitamin D for 24 weeks (Fig. 3a). Our findings suggest vitamin D insufficiency does not potentiate additional weight gain in lean or obese mice compared with sufficiency. Furthermore, no effect on BMI was observed in highly supplemented lean or obese mice, which is consistent with human studies that have attempted to promote weight loss through vitamin D supplementation [7–9, 39]. However, in our study, mice with high supplementation had serum 25-OH and 1,25-(OH)_2_ vitamin D concentrations similar to mice given standard amounts, which may indicate that these mice did not receive high enough supplementation to affect BMI. Indeed, evidence that greater supplementation might affect BMI was recently reported by Marcotorchino et al. [40], where 15,000 IU/kg chow given to mice prevented weight gain during a high fat diet. Mice in that study were only 6 weeks of age at the start of the experiment and not obese at baseline, yet these findings may indicate a necessary level of supplementation to see an effect.

Despite the lack of an effect of higher supplementation on BMI or serum vitamin D metabolites, our lean mice supplemented with 4000 IU/kg chow had greater caloric consumption over the length of the study (14.6 ± 1.7 kcal/mouse/day versus 11.8 ± 1.4 for 1000 IU and 12.3 ± 1.7 for 125 IU, ****p < 0.0001 for both, Fig. 3b). The inference from these data is that the greater food consumption was offset by higher metabolism, perhaps suggesting a weight loss strategy involving caloric restriction might be improved by vitamin D supplementation. Interestingly, Shapses et al. were unable to discern an effect on weight loss in humans with a dual vitamin D supplementation and caloric restriction protocol [41]. But it is possible that the 2500 IU vitamin D_3_/day supplementation strategy was not equivalent to the higher level of supplementation in our mice.

Overall, our findings that vitamin D insufficiency had no effect on BMI in our mice run contrary to findings by Bastie et al. [14] and to a lesser extent Liu et al. [13], where diet induced vitamin D deficient mice were resistant to “western” and high fat diet induced weight gain, respectfully. However these studies induced vitamin D deficiency concurrently with alterations in diet. Whereas in our study the mice had been fed a HFD for four months prior to initiation of the vitamin D intervention. Additionally we believe the age at which mice were made insufficient/deficient may also be an important factor. We observed that the average length of our obese mice at 48 weeks of age was significantly longer than lean mice (105.5 ± 1.3 cm versus 100.6 ± 2.3 cm, ****p < 0.0001, Fig. 3c). We found no length differences between vitamin D groups, which would be expected as mice have likely stopped growing by the time supplementation was adjusted. However, both Bastie et al. and Liu et al. varied vitamin D supplementation before 3–5 weeks of age which may have lead to numerous impacts on the development of the mice not seen in our study since variations in vitamin D supplementation occurred after maturity. Interestingly, although our study is the first report of high fat feeding leading to increased length in mice, several human studies have found trends between higher BMI and height in adolescents [42–44].

### Serum 25-OH vitamin D insufficiency does not modulate body fat, lean mass or bone mineral density in either lean or obese mice

**Fig. 4:**
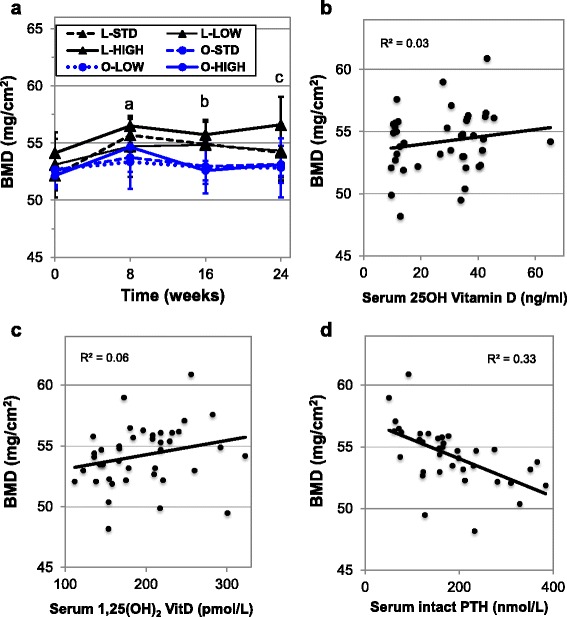
Impacts on bone mineral density and relationship with serum biomarkers. The effects of high fat and low fat diet containing varying levels of vitamin D on bone mineral density were analyzed using DEXA **a** No statistical differences observed between vitamin D groups. Differences between all lean and obese mice were significant for bone mineral density (^a^ *p* = 0.004; ^b^ *p* < 0.0001; ^c^ *p* < 0.008). Bone mineral density was not correlated with serum 25-OH **b** or 1,25-(OH)_2_ vitamin D **c**, but was correlated with serum intact PTH (R^2^ = 0.32, ****p < 0.0001) **d**

**Fig. 5:**
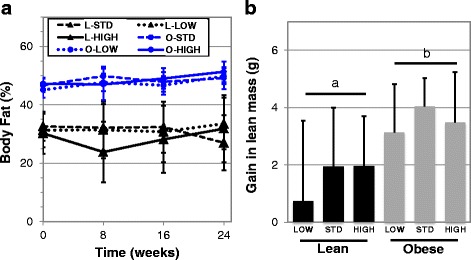
Analysis of body composition of lean and obese mice given low, standard and high vitamin D supplementation. The effects of high fat and low fat diet containing varying levels of vitamin D on body fat % **a** and the gain in lean body mass over 48 weeks **b** were analyzed using DEXA. No statistical differences observed between vitamin D groups. Differences between all lean and obese mice were significant for body fat % (at all time points, ****p < 0.0001) and gain in lean body mass (a versus b, **p = 0.002)

Vitamin D is canonically recognized as being important for bone health, and more recently for impacts on body fat and lean mass, yet the impacts of chronic insufficiency on these parameters have not been elucidated. Therefore, we performed dual X-Ray absorptiometry (DEXA) analysis at baseline and every two months thereafter on our mouse cohorts (Figs. 4 and 5). Surprisingly, despite 6 months of vitamin D insufficiency, neither lean nor obese insufficient mice exhibited lower BMD compared to sufficient mice (Fig. 4a). These findings are in general agreement with Fleet et al. [32], with the exception of lower BMD seen in a group receiving 25 IU vitamin D_3_/kg chow group, for which supplementation was below our lowest tested group. Interestingly, high supplementation failed to increase BMD in mice as well, although we note this may be due to the inability of this level of supplementation to substantially increase serum levels of 25-OH vitamin D or the possibility that we were underpowered to observe this effect. Furthermore, we did not identify association between BMD with either serum 25-OH vitamin D (Fig. 4b) or 1,25-(OH)_2_ vitamin D (Fig. 4c), but there was a negative correlation between BMD and serum intact PTH (r^2^ = 0.33, ****p < 0.0001 Fig. 4d). This difference remained significant when controlling for BMI of the mice (**p = 0.0014, data not shown) and reflects the key role of PTH in bone homeostasis [45].

However, obese mice exhibited lower BMD at all time points except at baseline (0 weeks: *p* = 0.17; 8 weeks: **p = 0.004; 16 weeks: ****p < 0.0001; and 24 weeks: **p = 0.008, Fig. 4a). Interestingly, in other mouse studies such differences in total BMD between obese and lean were not observed, yet differences in bone density were observed when utilizing μCT [46–48]. One key distinction between our study and the other reports is that the length of nearly 48 weeks in our study that may have provided enough time for obesity related-effects on BMD to become manifest. It is also noteworthy that higher supplementation was not able to counteract the effects of obesity. However, as higher supplementation did not significantly affect 25-OH or 1,25-(OH)_2_ vitamin D concentration, the possibility remains that supplementation beyond 4,000 IU/kg chow may rescue obesity related bone deficits.

Our data reveal that vitamin D insufficiency does not modulate body fat percentage (Fig. 5a) or lean tissue mass (Fig. 5b) in lean or obese mice. Yet, we did detect a larger gain of lean mass in obese mice versus lean mice over the 48-week period (**p = 0.002, Fig. 5b). Interestingly, no effects were seen in mice receiving higher amounts of vitamin D_3_ in chow contrary to observations in several human studies [7, 49, 50], but not all [33]. This discrepancy may be due to a differing range of supplementation levels, or that vitamin D may have roles unique to human physiology.

## Conclusions

We have established a model of vitamin D insufficiency in lean and obese mice by reducing vitamin D_3_ supplementation in chow. Obese mice exhibited a slower rate of decline in serum 25-OH vitamin D and modestly higher levels at equilibrium. No differences in serum 1,25-(OH)_2_ vitamin D, intact parathyroid hormone (PTH) or serum calcium were observed between lean and obese mice within the same vitamin D supplementation group. Yet obese mice exhibited lower serum calcitriol, higher serum PTH, and lower bone mineral density (BMD) than did lean mice. It also appears that 25-OH vitamin D concentrations between 10 to 40 ng/ml did not correlate with body weight or body fat in mice, although higher supplementation in mice fed a standard diet correlated with increased food consumption weight or lean/fat mass. The increase in caloric consumption without a concurrent increase in energy storage implies a heightened metabolism, which, if coupled to a calorically restricted diet may prove to be an effective strategy for weight loss.

## Abbreviations

BMI: Body mass index
DEXA: Dual Emission X-Ray Absorptiometry
iPTH: Intact parathyroid hormone
μCT: Micro computed tomography
VDR: Vitamin D receptor
